# Quantifying hormones in exhaled breath for physiological assessment of large whales at sea

**DOI:** 10.1038/s41598-018-28200-8

**Published:** 2018-07-17

**Authors:** Elizabeth A. Burgess, Kathleen E. Hunt, Scott D. Kraus, Rosalind M. Rolland

**Affiliations:** 1 Anderson Cabot Center for Ocean Life, New England Aquarium, Boston, 02110 USA; 2 Center for Bioengineering Innovation, Northern Arizona University, Flagstaff, AZ 86011 USA

## Abstract

Exhaled breath analysis is a non-invasive assessment tool that has shown promise in human diagnostics, and could greatly benefit research, management, and conservation of large whales. However, hormone assessment of whale respiratory vapor (blow) has been challenged by variable water content and unknown total volume of collected samples. To advance this technique, we investigated urea (a compound present in narrow range in circulation) as a normalizing factor to correct for blow sample concentration. Normalized progesterone, testosterone, and cortisol concentrations of 100 blow samples from 46 photo-identified North Atlantic right whales (*Eubalaena glacialis*) were more biologically relevant compared to absolute estimates, varying by sex, age class, or individual. Progesterone was elevated in adult females compared with other cohorts and highest in one independently confirmed pregnant female. For both sexes, testosterone was two-fold higher in reproductively mature whales but studied adult females showed the widest variation. Cortisol was present in relatively low concentrations in blow and demonstrated variation between individual whales, suggesting potential for studies of individual differences in adrenal activity. Incorporation of methodologies that normalize sample concentration are essential for blow hormone analysis of free-swimming whales, and measurement of urea could be used to optimize non-invasive physiological assessment of whales.

## Introduction

Exhaled breath analysis is an emerging technology used for diagnostic testing in human health^1–3^, and also holds promise in advancing physiological research of large whales^4^. Studies in humans have shown that the breath matrix contains valuable biomarkers, including proteins, steroids, lipids, cytokines, electrolytes, nucleotides, and urea^5–8^, due to a diffuse exchange with blood at the pulmonary alveolar membrane interface and the expulsion of airway lining fluid^3,6^. Analyzing biomarkers in breath has been explored for humans as a convenient and repeatable clinical procedure without harm or discomfort to patients^1^. Similarly, the appeal of investigating respiratory vapor of whales (blow)^4,9,10^ is based on its non-invasive approach and potential frequency of sample collection in a highly mobile species, since whales come to the surface of the water to forcefully exhale and take in oxygen. Whales breathe in large tidal volumes with extreme efficiency due to two layers of pulmonary capillaries for maximal exposure of lung air to blood circulation^11,12^. Moreover, the respiratory and digestive tracts of cetaceans are separated to a greater extent than other mammals, such that the larynx extends up to the nasal cavity rather than opening into the throat (cf. human breath analysis, where salivary contamination of the sample is a concern^6^). Due to the unique respiratory anatomy of cetaceans, there is potential that important physiological insights could be derived from whale respiratory vapor measurements.

The need to develop technologies for physiological assessment of free-swimming whales is driven by increasing anthropogenic pressures and ocean industrialization^13^. Measurable physiological biomarkers could afford managers insights into sub-lethal effects on health and reproduction^14^, potentially *before* deleterious population consequences occur^15^. To date, hormone analyses have provided valuable information on many aspects of cetacean physiology in the wild, including reproductive maturity, pregnancy status, metabolism and stress responses^16–24^, as well as possible cause-and-effect relationships^18,25–28^. These previous hormone studies have either relied on fecal samples collected opportunistically after defecation or blubber samples collected using biopsy darting. Despite the significant contribution of these sampling methodologies, there remains a need for a technique that permits repeated sampling of a targeted individual whale, throughout seasonal movements, life history changes, and fasting periods. Furthermore, unlike feces and blubber that accumulate hormones over hours or days^29^, the hormone content in blow may reflect more rapid and short-term acute responses^30^ associated with a particular event or stressor. As a more nascent sample type for hormone analysis, cetacean respiratory vapor has been shown to contain detectable levels of steroid (e.g., testosterone, progesterone, estradiol, estrone, cortisol) and thyroid hormones (e.g., thyroxine and triiodothyronine)^4,9,31–34^, and a vast number of volatile and nonvolatile compounds^34–36^, in addition to DNA from both the individual whale^37–39^ and respiratory microorganisms^10,40^.

For blow hormone analysis to become a useable diagnostic tool for large whales, hormone measures in the blow matrix must be quantifiable and resulting data must be shown to be physiologically valid. A recent study using samples collected from aquarium beluga (*Delphinapterus leucas*) demonstrated that testosterone and progesterone concentrations in respiratory droplets were correlated with blood concentrations^33^, showing physiological concordance between blow and the biological matrix most traditionally used in diagnostics. Researchers used volumetric measures to quantify blow hormone concentrations of belugas under controlled conditions that enabled sample collection of relatively undiluted respiratory droplets from multiple exhalations. Although this study achieved critical steps in the development of blow hormone analysis, the analytical techniques developed for small cetacean species in captivity (e.g., beluga^32,33^, bottlenose dolphin *Tursiops truncatus*^41^) or under restraint in the wild^32,33^ are not transferrable to free-swimming cetaceans, especially large whales. No studies to date have been able to determine an accurate and reliable volumetric measure of a blow sample collected from a whale at sea^4,9,36^. Quantifying hormones in large whale blow is challenging because respiratory droplets are markedly diluted by high and variable amounts of water vapor [determined by a whale’s ventilatory rate, the saturation of the exhaled air, the rate of condensation in the environment, and/or rapid evaporation of diffuse tiny droplets on the sampling surface] and a high potential for seawater contamination of a sample, when the whale breaks the surface of the water for exhalation. As a result, the total amount of respiratory fluid is highly variable and difficult to quantify in field-collected samples; and thus, concentrations of hormone in whale blow samples cannot reliably be standardized per unit volume (or mass).

There is a need to investigate a suitable independent biomarker present in exhaled whale blow that could be used as a reliable dilution indicator to correct for the amount of respiratory fluid collected, and standardize measurements (sample normalization). The most commonly measured biological fluid requiring normalization methods is urine because urinary solute concentrations vary greatly depending on various physiological factors such as water intake^42,43^. A widely accepted approach for urine volume correction is to express hormone metabolite levels relative to a reference analyte (e.g., creatinine) that is inherently present in all samples and reflects sample concentration^42,43^. In human exhaled breath studies, urea has been the most widely utilized indicator of specimen dilution^3,6,44–46^ because it has relatively low variation in circulation, it is a small molecule that readily diffuses between the plasma and airway fluid, and has a low volatility^30,47–49^. Given these properties, it should be possible to estimate the dilution of collected respiratory fluid by measuring the absolute quantity of urea in the sample^49,50^ (a diluted respiratory sample would have a lower urea concentration and vice versa), and then normalize hormone content by calculating the ratio of hormone to urea; reporting results as ng/mg urea. This approach could correct for seawater contamination, the amount of respiratory fluid collected, and/or individual variation in a whale’s exhalation volume; and ultimately, would permit comparisons of relative concentrations of hormones in whale blow samples.

Since blood sampling and standard endocrine validations (e.g., pituitary hormone challenges, radiolabeled hormone infusions) are not feasible for free-swimming whales^14^, testing the validity of this approach will depend upon verifying that normalized hormone concentrations in whale blow vary as expected with differing known physiological states^16,17^. The North Atlantic right whale (*Eubalaena glacialis*) is an ideal study population to develop and physiologically validate this method. North Atlantic right whales (hereafter, right whales) remain one of the most endangered large whales^51^ with the current population estimated at less than 500 individuals^52^. This population has been consistently monitored since 1980^53^, and the long-term North Atlantic Right Whale Identification and Sightings Database – grounded on the ability to identify most whales in the population – holds immense amounts of data on individual whales including the birth year (for whales sighted as calves), sex, calving history, habitat-use patterns, and the impact of human activities^54^. Moreover, long-term studies on fecal hormones in right whales have yielded extensive information on the endocrine patterns expected for various reproductive states in this species^16,17,24–26^. Having independent knowledge of the physiological status of individual whales (particularly, sex, age class [juvenile/adult], and reproductive status) allows biologically meaningful patterns in whale blow hormone results to be confirmed with *a priori* predictions.

Here, we leveraged four decades of dedicated right whale research to advance the quantitative analysis of hormones in whale blow. Our objectives were to: i) determine the presence of urea in whale blow and validate an assay protocol for reliable urea measurement in whale blow samples; ii) examine the variation of urea in whale blow samples to assess its potential as an informative dilution indicator for normalizing hormone concentrations in field-collected samples; iii) determine whether normalized progesterone, testosterone and cortisol concentrations in blow samples reflect endocrine profiles expected for whales of different known life history states; and iv) evaluate whether sample normalization methods compared to absolute (non-normalized) estimates better distinguished biologically relevant patterns for blow hormone analysis of large whales.

## Results

**Figure 1:**
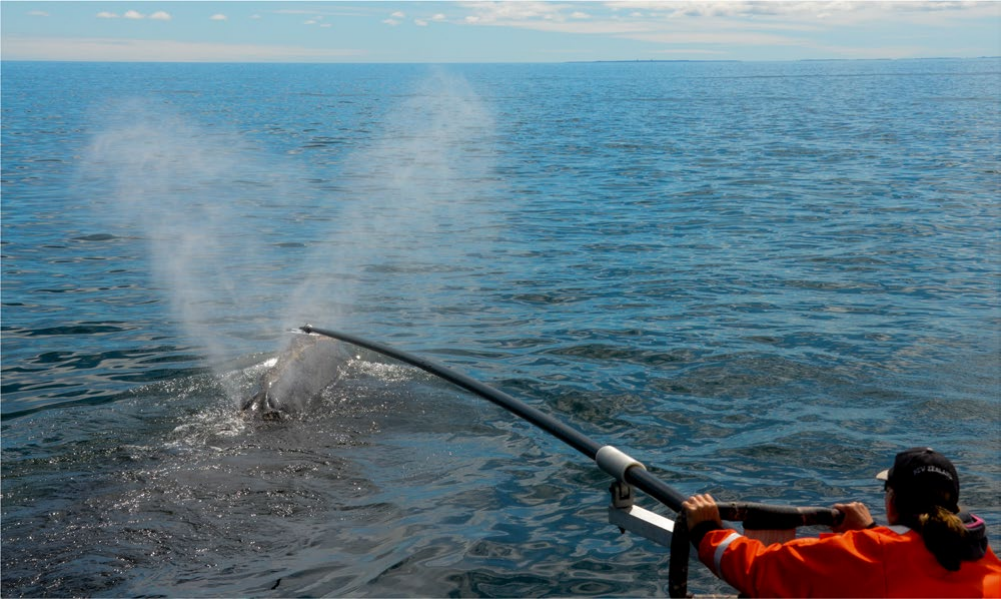
Collecting respiratory vapor from a North Atlantic right whale, using a polystyrene dish fastened to the end of a 9.75 m long pole and positioned above the exhaling blowholes [Photo taken under permit - refer to Methods].

A total of 100 blow samples were collected from 46 individual North Atlantic right whales over eight days (Fig. 1). Whales were all identified individuals of known sex and reproductive state (ages ranged from one to over 37 y.o.), except for one identified juvenile of unconfirmed sex (5 y.o.). Of the males sampled (n = 30 individuals), 13 were juveniles and 17 were adults (although a single sample from one adult male had zero detectable analytes). Of the females sampled (n = 15 individuals), eight were juveniles and seven were adults, comprising five resting females (i.e., non-lactating and not re-sighted with a calf), one lactating cow with an attendant calf, and one confirmed pregnant female (sighted the following year with a neonate calf). During each sampling encounter, a single sample was collected from an individual right whale. However, after photo-identification, we determined that repeat samples were collected from 24 whales. We also opportunistically collected a fecal sample from the pregnant female, and subsequently performed fecal progesterone analyses^16^ for a matched comparison with two blow samples collected from this female.

We successfully collected a blow sample from 74% of approached whales, with an average of 13 ± 10 samples collected per day at sea. Our sampling success rate significantly improved with experience (χ^2^ = 27.53, df = 7, *P* < 0.001), such that in our last two days of blow sampling, we sampled 94% and 100% of whales approached (n = 32 and 11 whales, respectively). There was no bias towards achieving better quality samples in the later days of collection (*r*_*s*_* = *−0.43, *P* = 0.67). The collection of a blow sample from an individual right whale was achieved on average within 5 ± 4 min (range <1 min and up to 23 min; 87% of samples were collected in <10 min). An *excellent* quality sample was typically collected within 4 ± 0.5 min, *good* samples within 7 ± 1 min, and *fair* samples within 10 ± 1 min. The number of exhalations collected per sampling event was mostly one (n = 75) or two exhalations (n = 16), but up to three (n = 7) and four exhalations (n = 2) could be collected from a targeted whale. Most samples were collected using a dish sampler (n = 74), with some samples collected using a nitex mesh sampler for comparison (n = 26).

### Variation in blow urea

**Figure 2:**
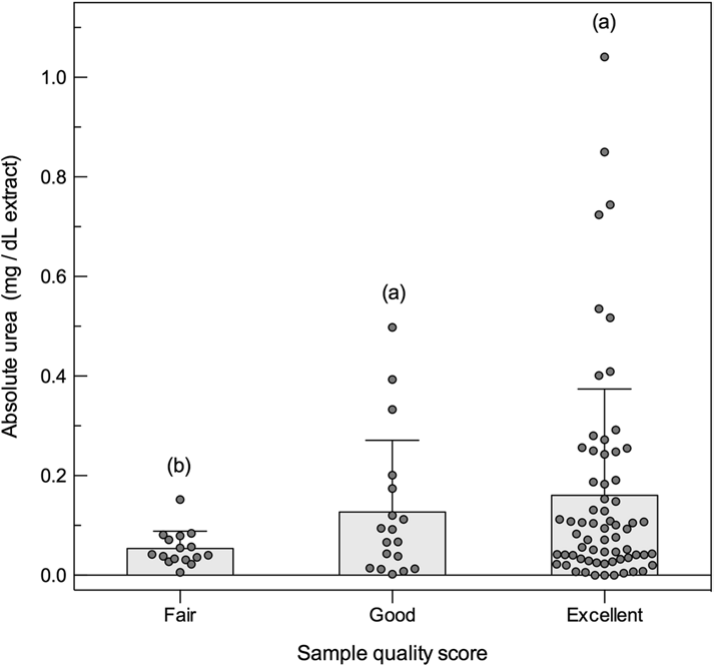
Absolute quantity of urea (mg/dL extract) in extracts of whale blow (total n = 100) matched the relative amount of respiratory fluid collected in each sample (subjectively graded as *fair*, *good*, or *excellent*), providing an indicator of sample dilution. Bar graphs represent means + SEM, with individual samples marked by a circle. Different letters denote a significant difference in urea between sample quality scores at *P* < 0.05.

The urea assay reported a small level of exogenous background noise from negative controls (i.e., non-zero results for blank samples) of nitex mesh samplers (0.13 ± 0.05 mg/dL), whereas negative controls of dish materials produced no assay interference (see Supplementary Table 1). Urea in whale blow was measureable in 95% of sample extracts, with five samples excluded from analyses due to potentially no biological matrix collected on the sampling device; these included 4% of samples collected on dish samplers that had zero detectable urea (n = 3) and 8% samples collected on nitex mesh (n = 2) that had urea levels (0.11 and 0.09 mg/dL) lower than the known assay interference for that sampling device. Absolute quantities of urea in whale blow extracts, corrected for assay interference, were on average 0.109 ± 0.016 mg/dL, ranging from 0.002 up to 1.041 mg/dL. Absolute urea quantities were not influenced by the different materials used in sample collection (Wald statistic = 2.10, df = 1, *P* = 0.15; dish: 0.06 ± 0.01 mg/dL, nitex mesh: 0.10 ± 0.04 mg/dL) but were associated with the quality score assigned to samples (Wald statistic = 9.45, df = 2, *P* = 0.01). The better quality samples had higher quantities of urea at 0.14 ± 0.02 mg/dL (n = 61 *excellent* samples, 0.11−0.18 mg/dL) and 0.12 ± 0.03 mg/dL (n = 18 *good*, 0.07−0.19 mg/dL), and inferior samples had the lowest urea levels at 0.03 ± 0.01 mg/dL (n = 16 *fair*, 0.01−0.08 mg/dL; *P = *0.04) (Fig. 2). Higher sample quality scores consistently predicted increased urea concentration in both sampler types (model interaction term: Wald statistic = 3.98; df = 2, *P* = 0.14).

### Variation in blow hormones

**Figure 3:**
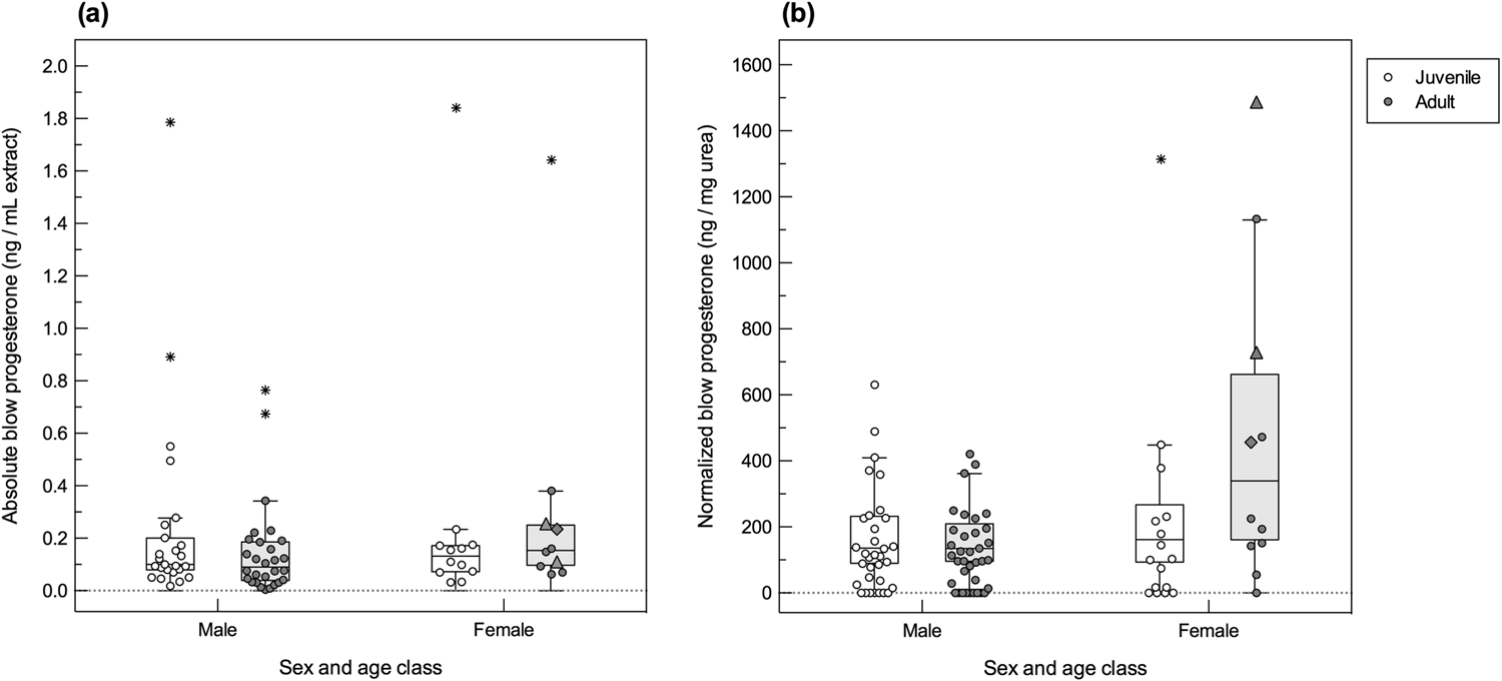
Variation in **(a)** absolute (ng/mL extract) and **(b)** normalized progesterone concentrations (ng/mg urea) in blow samples from North Atlantic right whales, according to different sex/age class and reproductive states. Individual samples are marked with open circles for juveniles and grey filled circles for adults. Samples from a confirmed pregnant female are highlighted with a triangle, and a sample from a lactating female is highlighted with a diamond. For boxplots, the line inside the box indicates the median value, the height of the box encompasses the distance between the 25th and 75th quartiles, and the whiskers delineate the highest and lowest values within 1.5 times the interquartile range. Extreme outliers in the dataset (>3 times the interquartile range) are marked with a star.

Progesterone in right whale blow was measurable in 78% of sample extracts, excluding 23% of dish (n = 17) and 19% of nitex mesh collected samples (n = 5) below the limit of detection. Normalized progesterone concentrations ranged between 13.9−1486.9 ng/mg urea. The best model evaluating normalized blow progesterone data had a high relative model weight (ω_*i*_ = 0.75) and included the effect of whale sex (LR χ^2^ = 12.88, df = 1, *P* < 0.001; Table 1). Individual whale identity was retained in the model but was not statistically significant (LR χ^2^ = 2.42, df = 1, *P = *0.12). Sex-related differences in normalized concentrations of progesterone indicated that female right whales had on average higher blow progesterone (365.1 ± 63.6 ng/mg urea, 259.5–513.7 ng/mg urea) than males (173.8 ± 19.5 ng/mg urea, 139.6–216.5 ng/mg urea; *P* < 0.001) (Fig. 3). Adult females (488.7 ± 123.1 ng/mg urea, 298.3–800.8 ng/mg urea) had nearly twice the progesterone concentration of juvenile females (260.3 ± 59.9 ng/mg urea, 165.8−408.6 ng/mg urea; *P* = 0.06), and higher concentrations than adult males (156.6 ± 24.1 ng/mg urea, 115.8–211.8 ng/mg urea; *P* < 0.001) and juvenile males (192.1 ± 30.5 ng/mg urea, 140.8−262.2 ng/mg urea; *P* = 0.002) (Fig. 3). Of the models tested, the highest AIC (indicating a worse fit) showed that the effect of sample quality did not explain the variation in normalized progesterone (∆AIC_*i*_ = 14.78). By contrast, absolute progesterone of blow samples (uncorrected for sample dilution) were best fitted by the model that included sampling device LR χ^2^ = 56.63, df = 1, *P* < 0.001) and had an AIC weight ω_*i*_ of 1.0; whereas all the other models had ω_*i*_ = 0, indicating that the differences in AIC_c_ distinguishing the best model from the others were substantial. In sum, absolute data (non-normalized; no urea correction) exhibited no trend with life history variables (Supplementary Table 2).

**Table 1:** Ranking of *a priori* models explaining the variation in normalized concentrations of (a) progesterone and (b) testosterone as reproductive hormones, and (c) cortisol as stress-related hormone, measured in the blow of right whales.

Model parameters	AIC_c_	df	ΔAIC_*i*_	*ω*_*i*_
**(a) Normalized progesterone:**				
**sex + individual whale**	**958.1**	**72**	**0.00**	**0.75**
sex + age class + individual whale	960.3	71	2.24	0.24
sampling device	967.9	73	9.80	0.01
individual whale	968.7	73	10.65	0.00
age class + individual whale	970.5	72	12.43	0.00
sample quality	972.8	72	14.78	0.00
**(b) Normalized testosterone:**				
**sex + age class + individual whale**	**974.1**	**79**	**0.00**	**0.84**
age class + individual whale	978.8	80	4.62	0.08
sex + individual whale	980.9	80	6.72	0.03
sampling device	980.2	81	6.01	0.04
individual whale	985.2	81	11.05	0.00
sample quality	987.3	80	13.09	0.00
**(c) Normalized cortisol:**				
**individual whale**	**698.9**	**86**	**0.00**	**0.33**
age class + individual whale	701.0	85	2.06	0.12
sex + individual whale	701.0	75	2.09	0.12
time of day + individual whale	701.2	85	2.25	0.11
repeat sample + individual whale	701.3	84	2.40	0.10
sampling duration + individual whale	701.5	85	2.61	0.09
sampling device	701.8	86	2.90	0.08
sample quality	702.7	84	3.78	0.05

Biological and sampling variables were all modeled as fixed effects, except for individual whale modeled as a random effect. Models were ranked based on Akaike’s Information Criterion adjusted for small sample size (AIC_c_)_._ The lowest AIC_*c*_ indicates the best model for each hormone measured (highlighted in bold). AIC_c_ weights (ω_*i*_) sum to 1 and indicate the relative likelihood of the model.

Sample normalization showed that the highest blow progesterone concentration (1486.9 ng/mg urea) was measured in the adult female (14 y.o.) that was independently confirmed pregnant. Two blow samples were collected from this pregnant female (progesterone content of the second sample taken 2 d later = 728.1 ng/mg urea,), and both samples had elevated progesterone compared to resting adult females (296.5 ± 129.4 ng/mg urea), and one lactating female (456.0 ng/mg urea). The fecal sample from this pregnant female (collected concurrently with the second blow sample) had elevated fecal progesterone concentrations (4827.4 ng/g dry feces) consistent with pregnancy (>1000 ng/g dry feces^16,24^). Two other samples with blow progesterone comparable to the pregnant female were collected from a 25-year-old female (1133.1 ng/mg urea), presumed to be non-pregnant (re-sighted a year later without a calf), and an outlier sample from a six-year-old nulliparous female (1313.9 ng/mg urea) (Fig. 3).

**Figure 4:**
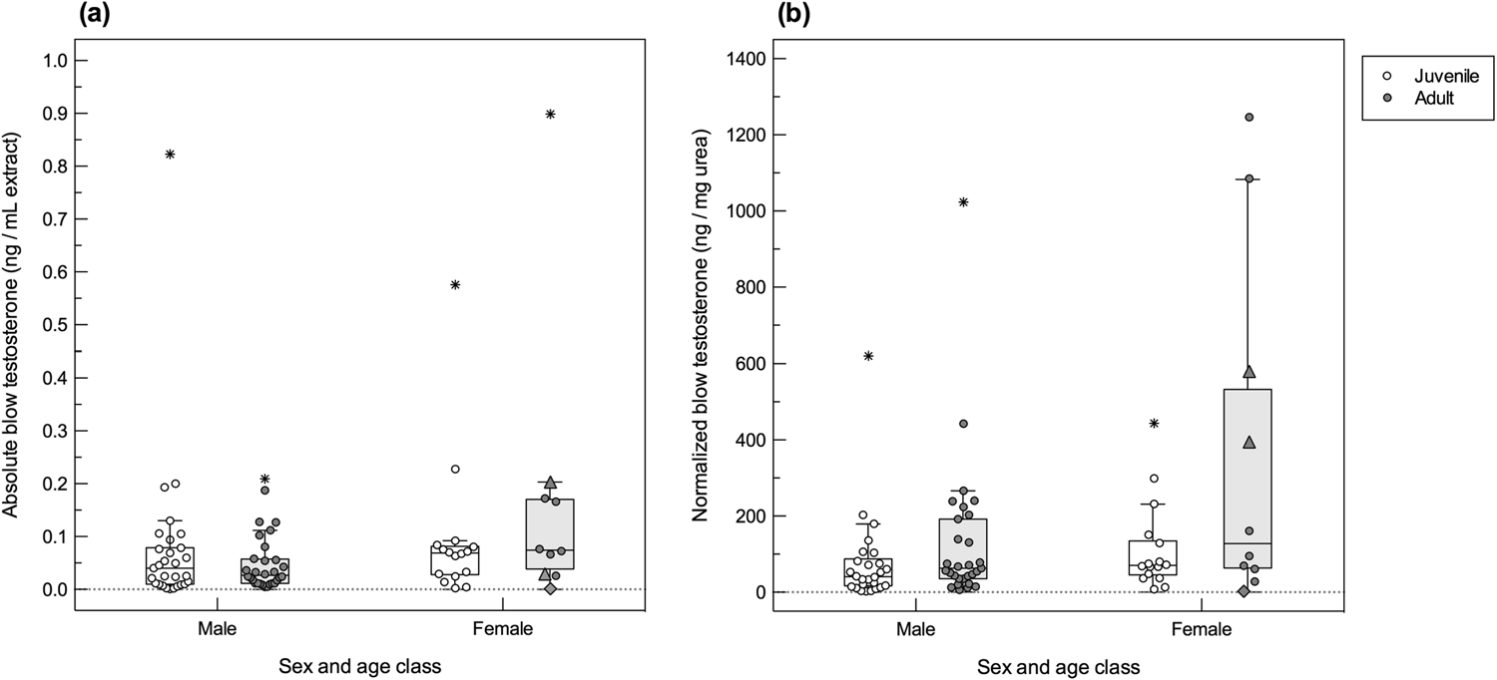
Variation in **(a)** absolute (ng/mL extract) and **(b)** normalized testosterone concentrations (ng/mg urea) in blow samples from North Atlantic right whales, according to different sex/age class and reproductive states. Individual samples are marked with open circles for juveniles and grey filled circles for adults. Samples from a confirmed pregnant female are highlighted with a triangle, and a sample from a lactating female is highlighted with a diamond. For boxplots, the line inside the box indicates the median value, the height of the box encompasses the distance between the 25th and 75th quartiles, and the whiskers delineate the highest and lowest values within 1.5 times the interquartile range. Extreme outliers in the dataset (>3 times the interquartile range) are marked with a star.

Testosterone in whale blow was measurable in 86% of sample extracts, excluding 15% of dish (n = 11) and 12% of nitex mesh collected samples (n = 3) below the limit of detection. Normalized testosterone concentrations in blow samples ranged between 2.2−1246.4 ng/mg urea. The top ranked model explaining variation in normalized testosterone data had a high relative model weight (ω_*i*_ = 0.84) and included the effects of whale age class (LR χ^2^ = 52.53, df = 1, *P* < 0.001) and sex (LR χ^2^ = 49.94, df = 1, *P* < 0.001; Table 1). In both sexes, reproductively mature whales had testosterone concentrations two-fold greater (209.1 ± 38.4 ng/mg urea, 145.9−299.6 ng/mg urea) than in immature individuals (98.3 ± 17.1 ng/mg urea, 69.9−138.4 ng/mg urea; *P = *0.01) (Fig. 4). More specifically, juvenile males had blow testosterone concentrations of 77.3 ± 16.2 ng/mg urea (51.2−116.7 ng/mg urea) and adult male levels were 134 ± 27.5 ng/mg urea (90.3−201.0 ng/mg urea; *P* = 0.06), with the highest blow testosterone concentration among males measured in an 11-year-old adult (1023.6 ng/mg urea). Adult females in this study tended to have wide variation in testosterone (372.2 ± 128.9 ng/mg urea, 188.8−733.8 ng/mg urea), which included the pregnant female (394.9 and 579.7 ng/mg urea), and levels were significantly higher than juvenile females (114.1 ± 16.2 ng/mg urea, 51.2−116.7 ng/mg urea; *P = *0.007) (Fig. 4). Sample quality scores did not affect normalized blow testosterone concentrations (∆AIC_*i*_ = 13.50), suggesting that normalization using urea effectively corrected the influences of variable sample dilutions at collection. By contrast, absolute blow testosterone data were best fitted by the model that included sampling device (ω_*i*_ = 0.95; LR χ^2^ = 61.85, df = 1, *P* < 0.001), and absolute (non-normalized; no urea correction) data exhibited a weak relationship with life history variables (all ω_*i*_ < 0.04; Supplementary Table 2).

**Figure 5:**
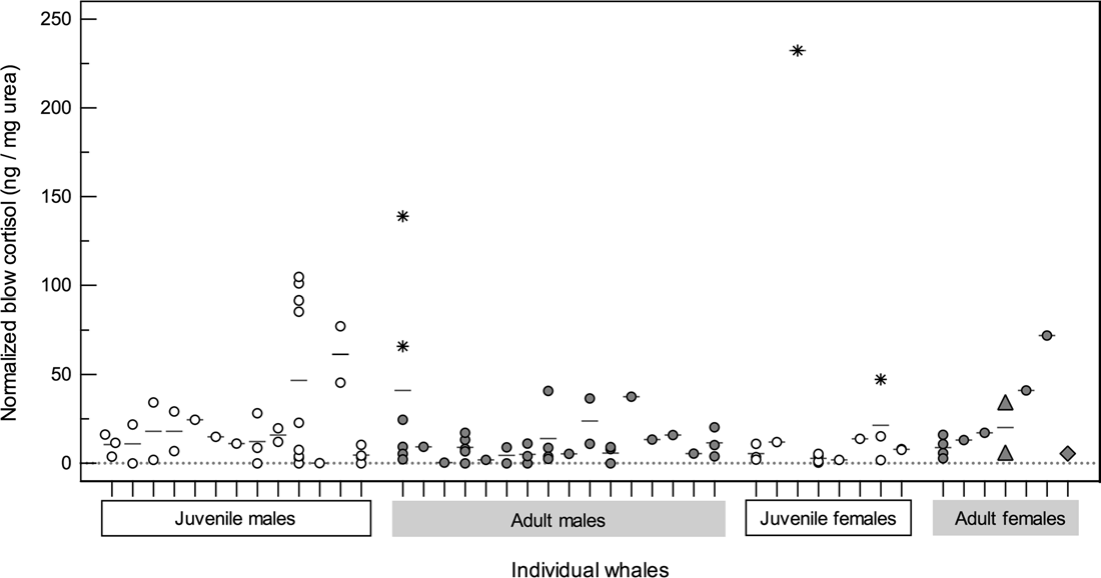
Variation in normalized cortisol concentrations (ng/mg urea) in blow samples from individual right whales (each column represents an individual whale), according to known sex/age class and reproductive state. Individual samples are marked with open circles for juveniles and grey filled circles for adults, with horizontal bars representing the mean value for each whale. Samples from a confirmed pregnant female are highlighted with a triangle, and a sample from a lactating female is highlighted with a diamond. Extreme outliers for their reproductive cohort (sex/age class) are marked with a star (>3 times the interquartile range).

Cortisol in whale blow was measurable in 86% of sample extracts, excluding 12% of dish (n = 9) and 19% of three nitex mesh collected samples (n = 5) that were below the limit of detection. Normalized cortisol concentrations in blow samples ranged between 0.1−232.3 ng/mg urea. For blow cortisol concentrations, a model with individual whale identity (LR χ^2^ = 47.52, df = 1, *P* < 0.001) had the most support relative to other *a priori* models (ω_*i*_ = 0.33) (Fig. 5). The next ranked models included whale life history traits (sex or age class) or various exogenous influences recorded during sample collection (time of day or repeat sample), and each of these models had evenly weighted support in accounting for the variation in observed blow cortisol among right whales (all ω_*i*_ ~ 0.10 and ∆_*i*_ ~ 2.0; Table 1). Similar to other hormone results, the worst-fitting model included sample quality score (∆AIC_*i*_ = 3.78; Table 1), again indicating that normalized blow cortisol concentrations were corrected for differences in collected sample dilutions. The best model for absolute blow cortisol data included sampling device (LR χ^2^ = 142.65, df = 1, *P* < 0.001) and had an AIC weight ω_*i*_ of 1.0, whereas all the other models had ω_*i*_ = 0.00, indicating this absolute (non-normalized; no urea correction) data exhibited no association with life history or stress-related variables (Supplementary Table 2).

## Discussion

This study represents the first assessment of whale blow analysis of free-swimming cetaceans at a population-level (~8–10% of the estimated population^55^), and including repeated sampling of individual whales. Although hormones have been previously detected in blow samples from large whales^4,9^, volumetric measurement and the ability to interpret differences in concentration have been thwarted by the highly variable and unknown water content of respiratory samples. Determining a methodology for sample normalization is fundamental because the major goal of physiological assessment is to examine the differences in analyte (hormone) quantities of two or more comparative samples. Here, we found that urea was detectable in whale blow, and that urea content in samples concurred with qualitative evaluations of the amount of respiratory fluid captured – showing that urea could be used to correct for variable sample dilutions, similar to its utility in human breath analysis^44,46,56^. Analyzing blow samples from known North Atlantic right whales permitted study of the physiological validation of this method for a large whale, and results showed that expressing blow hormone concentrations as a ratio to urea elucidated biologically relevant endocrine profiles that matched expectations for life history stages in this species. Differences in blow hormone/urea ratios were influenced by whale traits (sex, age, and reproductive state) known *a priori* to affect physiological state^16,17,24^, and were less associated with sampling artifacts, especially sample quality. The results of this study support the measurement of urea to optimize whale blow hormone studies.

A commercially available urea assay validated in this study was sensitive enough to measure urea in most right whale blow samples (0.01–1.0 mg/dL extract), including optimization of assay protocol for smaller sample volumes. Urea is an end-product of protein metabolism by the liver^57^, and is usually maintained at near constant rates in the blood^47^. Published data on urea concentrations in serum of deceased fin whales (*Balaenoptera physalus*) (99–184 mg/dL of serum)^58^ and living bottlenose dolphins (83–172 mg/dL of serum)^59^ verify that urea occurs within a relatively narrow concentration range in cetacean circulation. Furthermore, an experimental study on bottlenose dolphins found that circulating urea concentrations in individuals were generally stable, and unaltered in response to acute stress exposure (Champagne, pers. comm.). Although the physiological mechanism of urea entering the respiratory droplets of whales is not precisely known, most blood solutes are hypothesized to enter the fluid lining the respiratory tract via passive diffusion^30,44^. During exhalation, droplet formation occurs in the lung airways, where high air velocities and turbulence are encountered, mediating the transport of nonvolatile solutes (including urea and hormones) from the lungs to the environment in trace amounts by a relatively small volume of respiratory droplets^60^.

Urea levels in whale blow samples correlated with qualitative assessments of the amount of respiratory fluid collected, providing evidence that urea could be an indicator of the relative quantity of blow matrix obtained. Increased quantities of urea (>0.2 mg/dL extract) were only measured in samples graded as better quality based on visible fluid on the sampling device. However, not all samples visually scored as the best quality yielded a high concentration of urea, likely because optimal blow collection (i.e., sampler positioned closer to blowholes) can be exposed to an increased risk of seawater dilution as the whale breaks the surface of the water to exhale. Poorly graded samples consistently yielded very low urea levels, since peripheral sampling positions around the blow cloud will always reduce the opportunity for maximal respiratory fluid collection. Nevertheless, poorer quality samples collected in this study (scored as *fair*) still produced biologically plausible data, once hormone measures were normalized against urea. Furthermore, when absolute hormone measures for sample extracts were not normalized for urea, none of the biologically relevant relationships with individual whale sex, age class and reproductive state were observed. These results support the investigation of urea as a credible independent biomarker of sample dilution that could be used to normalize hormone concentrations in whale blow.

Sample normalization has not previously been applied to blow hormone analysis of cetaceans. Given that volumetric comparisons are not possible for large whale blow samples due to numerous uncontrolled processes (e.g., seawater contamination, ventilation rate, condensation rate, or evaporation rate), this approach appears to have merit in providing more reliable hormone quantification of whale blow and enabling comparative assessment. When hormone measures were adjusted using the reference analyte of urea, whale blow concentrations of both reproductive hormones (progesterone and testosterone) reflected biological differences in sex and/or age class. Similarly, normalized cortisol concentrations revealed differences in individual whales across the studied population. Overall, normalized concentrations for all hormones measured in this study were not associated with external factors linked to sampling artifacts, suggesting that normalization methods were effective for overcoming the inherent dilution variability in different samples of whale blow and potentially improving the data quality. By contrast, absolute measures (raw non-normalized values) of all three hormones did not distinguish significant patterns with any biological variables that are known to be associated with physiologic changes in right whales; and instead, absolute (non-normalized) data aligned with non-biological factors related to the specific device used to collect the sample. The results indicated that the normalization of hormone concentrations in field-collected samples from large whales is important for furthering our understanding of this sample matrix and promoting reliable determination of the true biological variability of hormones in whale blow.

Progesterone was elevated in known reproductively mature female right whales, and the highest progesterone concentration in the study was measured in an adult female confirmed pregnant. A repeat blow sample from this pregnant female also had normalized progesterone levels 1.5-fold above resting adult females, and high fecal progesterone concentration in this female demonstrated congruence between blow and a well-studied hormone matrix in right whales^16,24^. Samples from an adult female (not re-sighted with a calf) and a nulliparous female classified as juvenile had levels within the progesterone range of the pregnant female. Although confirmed pregnancy samples were limited to one whale and inter-sample variability was evident, the progesterone signals of all whale blow samples adjusted according to urea quantities were generally consistent with female reproductive states associated with significant increases in circulating progesterone. Elevated blow progesterone in females presumed non-pregnant in this study may be reflective of reproductive cycle stage, since longitudinal studies on captive belugas have shown that blow sampling can be sensitive enough to detect increases in progesterone with luteal activity^33^. In the case of the younger female (6 y.o.), precocious sexual maturation as early as five years of age has been recorded for female right whales^61^. Increasing sample sizes will aid in establishing the concentration ranges in blow that are associated with pregnancy, reproductive cycles, and lactation in female right whales. The reproductive state of an unknown whale should ideally be distinguished from a single sample. However, similar to recommendations for hormone measurements of blood^62^, repeated blow sampling of individual whales may be necessary to reliably discriminate between pregnancy and a non-conceptive cycle (luteal phase). Additional research is required for blow sampling to better delineate within-subject variability in blow samples, and to provide diagnostic capabilities for free-swimming right whales.

Sexually mature whales tended to have higher blow testosterone levels than immature whales across both sexes. Adult males had twice the testosterone in blow compared to juvenile males, similar to patterns in right whale fecal hormones^16,24^. However, the relative increase in blow testosterone with maturation in males was smaller than that observed among females in this study. Higher testosterone concentrations in adult females were largely driven by three whales, including the confirmed pregnant female. Elevated testosterone has also been measured in blow samples collected from pregnant belugas held in captivity^33^, in fecal samples from pregnant right whales^24^ and killer whales (*Orcinus orca*)^28^, and a longitudinal study observed pregnancy associated increases in androgen production in captive killer whales^63^. In the present study, the highest testosterone concentration measured in a male was nearly five-fold higher than most adult male samples and comparable in magnitude to maximal female levels, indicating a potential for greater elevations in blow testosterone among males. Given the degree of overlap in concentrations between adult males, juvenile males, and females, additional sampling across different seasons and/or locations is required for better resolution of testosterone patterns in blow samples of males.

Cortisol concentration in blow was low compared to progesterone and testosterone, which agrees with a typical glucocorticoid concentrations in other sample matrices from this species (e.g., feces and baleen^16,17,64^) and most mammals^65^; i.e., baseline glucocorticoid levels are maintained within a generally low and restricted physiological range in circulation^65^. In contrast to the sex hormones, cortisol concentrations in blow were more strongly explained by individual variation between whales rather than life history states. Such inter-individual variation in cortisol could be indicative of the reactivity of the hypothalamic-pituitary-adrenal axis of individuals in triggering the production of glucocorticoids^66^, suggesting that blow as a matrix may reflect acute changes in endocrine responses. Similarly, a study on aquarium and wild-caught belugas found evidence of individual differences in blow cortisol measures, potentially due to response to out-of-water sampling, individual experience, and social dynamics^32^. The duration of the sampling attempts in this study did not prominently influence blow cortisol levels in right whales. Blow samples were obtained within a relatively short timeframe (all ≤23 min), and the lack of a detectable adrenal response may indicate that the whales were not disturbed by the vessel presence and/or a longer time-course for hormone signals in whale blow. A range of factors showed some evidence of association with blow cortisol, including whale life history traits (sex and maturity), time of day, and repeated sampling of individual, and each of these variables may contribute to observed inter-individual differences of whales; further exploration across different seasons and/or habitats or implementing experimental study design may be required for better interpretation of these factors. Some of the individual variation between whales may be due to factors not controlled for in this study, such as whale behavior prior to approach (e.g., feeding, social interaction), and/or other disturbances in the area (e.g., presence of other vessels or underwater noise levels^26^). Future studies investigating blow samples from whales of compromised health, poor body condition, or exposed to a known stressor (e.g., fishing gear entanglement)^17,25,26^ will allow us to better understand changes in adrenal activity as reflected in whale blow.

For blow sampling to be a feasible approach for hormone analysis in any free-swimming cetacean, the yield recovered from a sample of blow should be sufficient to allow for multiple analyses, i.e., measure a combination of sex and/or stress hormones as well as a compound for sample normalization from the same sampling event. A sample collected within 1 m of the whale’s blowholes yielded enough extract to perform four single-analyte assays, which involved sample extract dilutions of 1:2 or 1:4 for progesterone and testosterone assays, and undiluted (neat) extracts for cortisol and urea assays. Alternative analytical technologies such as liquid chromatography tandem mass spectrometry (LC/MS) can allow for simultaneous determination of multiple analytes from a single sample volume^9,31,34,35,41^. However, LC/MS systems previously used for measuring steroid hormones in whale blow had a reduced analytical sensitivity compared to immunoassay technologies (e.g., reported quantification limits of 500 pg/mL for progesterone, testosterone and cortisol^31,41^), and entail higher instrumentation costs^67^. The continued application of new technological advancements on cetacean samples will help optimize limited sample volumes, and subsequently cost-effectiveness; for example, nanospray-LC/MS with a limit of detection of 7–8 pg/mL for testosterone or progesterone was recently trialed for hormone analysis of whale blubber^68^. Nonetheless, given the naturally low concentration matrix and dispersion of respiratory fluid at exhalation, researchers should aim to maximize the amount of sample collected to be able to conduct analyses at optimal performance. Future studies will also benefit from using one type of sampling material that produces minimal non-specific binding (e.g., polystyrene dish samplers), and researchers must determine the degree of exogenous assay interference from sample processing by including negative controls^69^.

This study presents the first effort to normalize blow hormone measures from swimming large whales at sea, demonstrating that normalized progesterone, testosterone and cortisol concentrations of field-collected blow samples were biologically relevant, varying by sex, maturity status, and between individual whales. Advancing the use of whale blow from hormone detections^4,9^ to hormone quantification (this study) is vital for intended physiological assessments of whale populations^13–15^. Incorporation of methodologies for sample normalization (such as using urea as the denominator) must be performed in quantitative physiological studies where the analyzed samples have markedly variable water content, and this issue is exacerbated for blow hormone analysis of free-swimming whales. The results presented here justify further study of biomarkers for volume correction of whale blow samples. Given a sufficiently sensitive assay, it is likely that any small and stable molecule in the airway lining fluid could be detectable and quantifiable in whale blow, and the authors encourage further exploratory analysis of other viable dilution factors (e.g., total protein, total lipid, specific gravity)^5–8,42,46^. We caution that for most biomarkers in respiratory fluid, analytical techniques and assays will likely be employed at or near their detection limits, leading to potential variability in sample data. Therefore, maximizing sample concentration, developing more sensitive analytical techniques, and protocols for sample quality control will greatly assist in improving reproducibility and the diagnostic value of whale blow analysis.

Similar to the advancements of exhaled breath analysis for humans^46,49^, this emerging technology for cetaceans will be augmented by progressive technical improvements, and represents a considerable advance for studying large whales. Whale blow analysis may enable researchers to non-invasively and repeatedly sample a selected individual free-swimming whale to gain endocrine information in near-real time. This method could make it possible to determine the sex and maturity state of a whale, and to record data on pregnancy, reproductive cycles, and adrenal stress responses of large whales with temporal and spatial detail that until now were infeasible. Continued validation testing and method refinement using samples collected from well-studied individuals, including cetaceans held in captivity for which serum and blow analytes could be compared to gain an understanding of the time course of physiological processes and the degree of association between matrices, will aid in the application of blow sampling to other whale species. Priority areas for research include ascertaining the mechanisms of particle formation of exhaled respiratory fluid in cetaceans; determining the variability of urea excretion rates both within and between individual whales; employment of whale blow sampling in longer-term studies to delineate reference ranges; development of inclusion criteria for samples and evaluating within-subject variability and sample reproducibility; and ultimately, achieving standard analytical procedures for endocrine profiling of whale blow. Sample normalization should be part of the process for quantifying hormones in blow samples from large whales at sea, and this approach holds promise as an informative tool for physiological assessment of free-swimming whales in a rapidly changing ocean environment.

## Methods

### Study species and sample collection

North Atlantic right whales were sampled in daylight hours (between 06:00 and 18:00) in calm seas (<3 Beaufort sea state) along the eastern Atlantic seaboard, where right whales congregate for seasonal feeding. Blow samples were collected using a sampling device (see below) fastened to the end of a carbon fiber pole (9.75 m long), which was mounted to a cantilevered pivot on the foredeck of an 8-m research vessel^4^ (Fig. 1). During sample collection, the vessel slowly approached an individual whale at idling speed on a gradually converging course to minimize disturbance to the whale. The sampling device was held ~3–4 m above the water and rotated skyward to avoid seawater contamination until there was a good chance of obtaining a sample. On anticipating surfacing behavior of the whale, the pole was extended and lowered to position the sampling device above the exhaling blowholes (0.2–0.8 m) to catch a portion of the aerosol droplets. To evaluate the efficiency of blow sample collection, we recorded the sampling outcome for every whale that was approached for a blow sample (sample successfully collected or no sample). Date, time, and location (latitude/longitude) of collection were also recorded. Field research on right whales was approved by the New England Aquarium’s Animal Care and Use Committee (IACUC) and carried out under the U.S. National Marine Fisheries Service permit number 14233 and Canada’s Department of Fisheries and Oceans permits under the Species at Risk Act.

#### Sampling devices

Two different sampling devices were used to examine the practicality of each material for collecting blow from free-swimming whales, and to provide a sampling device comparison. Both devices had passed prior laboratory validations^69^, but each sampling material had different physical qualities for collecting a volume of sample. The preferred device for optimal analytical precision^69^ was a sterile polystyrene dish (25 cm × 25 cm; Corning® bioassay dish CLS431111, Sigma-Aldrich, St Louis, MO, USA; ‘dish’ hereafter) (Fig. 1). The second sampling device was a single-ply of nylon 110 µm mesh (cut to 30 cm × 30 cm; Nitex nylon, Elko Filtering, Miami, FL, USA; ‘nylon mesh’ hereafter) stretched over a clean plastic framework, which had previously been used in published studies as a collection material for cetacean blow^4,32,33^. In preparation, nylon mesh was thoroughly washed before use to remove potential interfering exogenous particles – using separate wash cycles of soapy water, distilled water, and 70% ethanol as previously described^69^.

#### Sample quality score

Every sample of whale blow collected was subjectively scored for quality, based on the proximity of the sampling device to the whale’s exhaling blowholes and the amount of visible blow droplets collected. Sample quality scores were: *fair* = sampler was in the exhaled vapor at >2 m above the blowholes, collecting diffuse fine droplets; *good* = sampler was 1–2 m above the blowholes, collecting coarse droplets covering <30% of sampling surface; *excellent* = sampler was <1 m above the blowholes, collecting coarse droplets across >30% of sampling surface. This qualitative score was recorded on the presumption that it characterized the amount of respiratory fluid collected on a sampling device^4^, with samples scored as *excellent* likely holding greater sample volume. If the sampling effort was not successful or poorly scored, we redeployed the sampler to collect from the same whale until it dived, recording the number of collection attempts (up to four blows collected for a given sample), and the revised quality score. Immediately after collection, the sampling device was placed in a protective zip-type bag, detached from the pole, and stored on ice packs in a cooler before being frozen at −80 °C upon return to shore (typically within 4 ± 0.2 h of sample collection). Previous testing has confirmed that steroid hormones are stable under these field storage conditions for at least 6 hours^69^.

#### Assigning reproductive state

Sampled whales were photographed to enable individual identification, based on unique markings such as callosity patterns and scars, and to obtain life history data using the North Atlantic Right Whale Identification and Sightings Database^55^. Photo-identification of individual whales was performed after conclusion of fieldwork by expert personnel using well-established protocols^54^. Whales were categorized as juveniles (1–8 y.o. and never calved) or adults (year before first calving or ≥9 y.o.)^70^. Pregnant females were confirmed by multiple sightings with a dependent calf in the year after sampling. This method of identifying pregnancy in females would not account for perinatal mortality, spontaneous abortion or undetected embryogenesis; i.e., we cannot rule out the possibility that some females re-sighted without calves may in fact have been pregnant the year prior.

#### Evaluation of other sampling influences

When an animal perceives a stressor, a typical physiological response involves a measureable increase in circulating glucocorticoids (including cortisol) within 5 minutes^65^. Blow sampling does not make contact with the whale; however, the use of an extended pole to collect the blow sample does necessitate a close vessel approach (ca 5–10 m) over a period of time, such that the duration of the sampling event and repeated sampling of an individual may influence the adrenal stress response of a whale – and ultimately, might affect cortisol concentrations in the collected blow sample. Therefore, we recorded the duration of sampling for each whale (number of minutes between initiation of the slow approach towards the whale and collection of the sample), as well as whether or not the identified whale had previously been sampled during the study period (categories of *first sample collected*, *repeat sample on same day*, or *repeat sample on a different day*).

### Sample analysis

#### Sample extraction

Obtaining a true volumetric measurement of respiratory vapor in each sample collected from a large whale was not possible due to various factors (see Introduction), especially unknown seawater contamination^4,9^ and air-dried sample potentially adhering to the sampling surface^69^. To process samples, we used methods validated by Burgess *et al*.^69^ for extracting hormones from low-volume samples collected on dish and nylon mesh devices. In brief, dish samples were extracted by pouring 50 mL of 100% ethanol (EtOH) onto the dish surface, which was then lidded and gently agitated on a plate-shaker for 30 min. This EtOH rinse was decanted into 25 × 125 mm borosilicate glass tubes and dried under compressed air for 24 h. Nylon mesh samples were extracted by pouring 80 mL of 100% EtOH over each mesh inside a 120-mL polypropylene jar. The jar was vigorously mixed on a plate-shaker for 1 h, after which the liquid was decanted into 25 × 125 mm borosilicate glass tubes. The nylon mesh component was centrifuged at 4000 *g* for 15 mins to separate additional liquid, which was added to the glass tubes. The zip-type bag that held the nylon mesh sample was also rinsed with 20 mL of 100% EtOH. The combined ~100 mL EtOH rinse was dried in glass tubes under compressed air for 24 h. All samples were reconstituted in 1.0 mL of dH_2_O (= total extract volume; N.B. extract volume does not relate to the original [unknown] sample volume of respiratory droplets collected at sea), and stored frozen at −80 °C until hormone analysis.

#### Urea analysis

Urea was investigated in whale blow because of its use as a normalization factor in several studies^49,50^, and to examine the assumption that urea amount reflects the blow concentration of samples collected from large whales. Other potential biomarkers for evaluating dilution, albumin and creatinine, were investigated in supplementary trials but these compounds had unsuccessful detectability in blow sample extracts when using commercial assay kits - and subsequently, further investigation of these compounds was halted to proceed with the development of urea analyses.

Blow sample extracts were analyzed for urea using a colorimetric detection kit (#K024-H1; Arbor Assays, Ann Arbor, MI) designed to quantitatively measure urea nitrogen in various sample types (including saliva, another fluid with low urea concentration). The urea assay is more sensitive to sample turbidity than the hormone assays; therefore, all samples were clarified before urea analysis via centrifugation at 5000 *g* for 10 min, followed by filtration of 100 uL aliquot of the resulting supernatant through a 0.22 µm pore membrane unit (#SLGVX13NL, Millex-GV hydrophilic PVDF membrane filter; EMD Millipore, Darmstadt, Germany) using a disposable Luer-Lock™ syringe (1 mL; #14-823-30, BD, NJ). For urea assay, nine standards (0.04–10.0 mg/dL; assay sensitivity = 0.01 ± 0.02 mg/dL) and clarified samples (undiluted) were loaded in duplicate as 30 uL volumes and mixed with kit reagents in a 96-well microtiter plate. This assay was performed at 60% volume (i.e., all reagent volumes were reduced to 60% of that stated in the manufacturer’s protocol) to minimize volume required from each blow sample; in-house testing verified that a 60%-volume protocol maintains good assay performance with acceptable accuracy and sensitivity (data not shown). Next, the plate was incubated at room temperature for 30 mins before reading the optical density at 450 nm. Urea concentrations were determined using a four-parameter logistic model based on the standard curve. Raw assay results for urea nitrogen were converted to urea by multiplying by 2.14, and expressed as milligrams of urea per deciliter of extract volume (absolute urea mg/dL extract).

#### Hormone analysis

Enzyme immunoassay kits (EIA; Arbor Assays, Ann Arbor, MI) were used for the quantification of progesterone (#K025-H1), testosterone (#K032-H1) and cortisol (#ISWE002) in all samples. Assay methods were performed according to manufacturer instructions (see http://www.arborassays.com), except that an additional low standard was included in each standard curve to increase the detection range, i.e., assay standard curve for progesterone ranged from 0.025 to 3.2 ng/mL (8 standards; assay sensitivity = 0.012 ± 0.008 ng/mL); for testosterone from 0.021 to 10.0 pg/mL (8 standards; assay sensitivity = 0.006 ± 0.006 ng/mL); and for cortisol from 0.0125 to 3.2 pg/mL (9 standards; assay sensitivity = 0.003 ± 0.002 ng/mL). For progesterone and testosterone assays, blow extracts were diluted at 1:4 with assay buffer (#X065; Arbor Assays). Some samples were re-assayed at 1:2 dilution to bring assay results nearer to 50% binding for best assay precision. Seventeen samples for progesterone and one for testosterone were below the limit of assay detection. For cortisol, blow extracts were analyzed undiluted; all had detectable cortisol. Each plate contained a configuration of standards, non-specific binding wells, maximum binding wells and controls run in triplicate (i.e., in duplicate at the beginning of the plate and singular at the end), and samples were assayed in duplicate. Hormone concentrations were determined using a four-parameter logistic model based on the standard curve. Raw assay results were expressed as nanogram of hormone per milliliter of extract volume (absolute hormone ng/mL extract) [N.B. extract volume was always 1.0 mL and absolute hormone values are not relative concentrations, since all samples were dried and reconstituted in 1.0 mL dH_2_O].

#### Assay quality control and verification

Since the assay kits used here were not designed for use with whale respiratory vapor samples, the suitability of each assay kit for measuring blow extracts was assessed. Parallelism and accuracy validation tests^71^ were performed using a pool of sample extract to ensure that antibodies and reagents recognized the targeted analyte in whale blow in a predictable manner and without interference (see Supplementary information). The binding of serial dilutions of blow extract (neat to 1:64) was parallel to the standard curve in immunoassays (progesterone: *F*_1,9_ = 3.06, *P* = 0.11; testosterone: *F*_1,8_ = 0.16, *P* = 0.70: cortisol: *F*_1,13_ = 0.63, *P* = 0.44; see also validations by Hunt *et al*.^4^) or colorimetric assay (urea: *F*_1,12_ = 2.31, *P* = 0.15; see Supplementary Figure 1a), indicating that substances in whale blow extracts do not interfere with antibody binding. All assays exhibited accuracy at their target dilutions (progesterone: slope = 1.14, *r*^2^ = 0.99; testosterone: slope = 0.83, *r*^2^ = 0.99; cortisol: slope = 0.72, *r*^2^ = 0.99; urea: slope = 0.99, *r*^2^ = 0.99), verifying reliable determination of analyte concentrations in right whale blow samples due to good mathematical accuracy across a range of concentrations from very low to high (see Supplementary Figure 1b; see also validations by Hunt *et al*.^4^). Additionally, seawater samples collected during field sampling were analyzed in each assay, with zero detectable hormone or urea measured.

To monitor precision and reproducibility in assays, high (~30%) and very low (~90%) concentration control samples were run on each plate (n <6 assays performed for each analyte). All assays were performed by the same person, and any sample with a coefficient of variation (CV) between duplicates of >10% was re-assayed^72^. For all assay types, the intra-assay CVs between sample duplicates were <8.2% (2.6 ± 0.3%), and the inter-assay CVs were <5.4% (2.9 ± 1.0%) and <10.5% (6.4 ± 1.4%) for high and low concentration controls, respectively. To quantify assay sensitivity, zero-standard replicates (n = 20 wells) were analyzed, with sensitivity calculated as the mean of assay results for zero-standard replicates ±2 standard deviations^72^.

#### Assay interference

Various materials used in the collection and processing of blow samples have been shown to introduce consistent low levels of assay interference^69^; therefore, we tested for exogenous interference in all assays for both sampling devices. As recommended by Burgess *et al*.^69^, blank materials of dish and nitex mesh were extracted and processed following the same procedure as a biological sample (n = 20 for each different sampler type), and then assayed for urea and all three hormones to achieve an estimate of background (spurious) measures in these negative control samples. Based on results (see Supplementary Table 1), sample concentrations for urea, progesterone, testosterone and cortisol were all adjusted for low and consistent background levels by subtracting the mean negative control concentration for that sampling device from the observed concentration. This correction helped to evaluate whether adequate blow sample had been collected for hormone analysis, since only those sample measurements greater than known assay interference for the sampling device were retained in the dataset (i.e., limit of detection). All analyte data are reported corrected for assay interference.

### Statistical analysis

The rate of success for collecting a blow sample from an approached whale during each day of fieldwork was analyzed using Chi-square analysis. Spearman rank correlation was used to investigate whether the quality score of samples improved with the number of days spent sampling. Data on whale identity, sex, and reproductive state were integrated with blow analysis results for all samples. Analyte data were all modeled in a generalized linear model (GLM) framework using a log-link function and gamma distribution, which better accounted for the right-skewed distribution of measured concentrations in blow.

To investigate urea as an indicator of the dilution of collected blow samples, we used a GLM to examine the quality score of samples [*fair*, *good* or *excellent*] and the type of sampling device [dish or nitex mesh] as explanatory variables of absolute urea concentration in blow extracts (ng/mL extract; response variable). An interaction term (quality score × sampling device) was included to consider the possibility that urea concentrations may not exhibit the same changing relationship across quality scores in dish and nitex mesh samplers. Given that urea is typically maintained in a narrow concentration range in the body^47^, samples with poorer quality scores were predicted to have consistently low levels of absolute urea per mL of extract to reflect lower volumes of respiratory fluid collected. Conversely, samples with better quality scores were predicted to have the highest levels of absolute urea per mL extract. Detecting these trends would indicate that urea content of extracts reflects the amount of respiratory fluid collected from a whale (i.e., urea is a meaningful dilution indicator), which is a fundamental validation before proceeding to test urea as a normalizing factor for the variable dilution of respiratory droplets. Based on the results, hormone assay results were normalized against the amount of urea in each extract, using the formula: blow hormone concentration_normalized (ng/mg urea)_ = absolute hormone_(ng/mL extract)_/absolute urea_(mg/mL extract)_. Thus, hormone concentrations of blow samples were quantified as nanogram of hormone per milligram of urea (ng/mg urea).

Hormone data were analyzed using generalized linear mixed models to allow both fixed and random components to be fitted to a model; in this case, individual whale (i.e., whale identity) was included as a random effect to account for individual-level variability. For models, we incorporated predicted explanatory variables that were known for each right whale and each sampling event, including whale sex and age class [juvenile or adult], the duration of the sampling event [binned as ≤3 min, 4–10 min or >10 min], time of day [hour integers, 6:00–18:00], sample quality score, type of sampling device, and sample occurrence for an individual whale [categories of *first sample collected*, *repeat sample on same day*, or *repeat sample on a different day*]. A set of competing *a priori* models were generated that tested different ways in which blow hormone concentrations – progesterone and testosterone as sex hormones (6 models), and cortisol as a stress-related hormone (8 models) (see Table 1) – could vary as a function of effects associated with life history traits and stress-related influences (i.e., biologically meaningful measures), or from non-biological variables associated with sampling artifacts (i.e., measures that might indicate sample concentration was not adequately standardized for dilution). NB: both dish and nitex mesh samples were retained in the dataset because ‘type of sampling device’ provided an additional and useful non-biological variable with which to evaluate the validity of hormone normalization results. We hypothesized that normalized hormone values (ng/mg urea) would demonstrate reliable quantification of hormone concentration in different whale blow samples, exhibiting stronger associations with biologically relevant factors than sampling artifacts. Finally, we conducted the same model analyses on the dataset of absolute hormone values (ng/mg extract) that were not adjusted for differing amounts of sample collected, and therefore, not standardized for true sample concentration (i.e., not relative data). Inclusion of model results for non-normalized hormone data permits comparison between normalized data outcomes and random expectations.

Akaike’s Information Criterion (AIC) with the adjustment for small sample size (AIC_c_) was calculated for each model and used to objectively rank the different models^73^. This approach weighs models by the amount of the variance explained and model complexity (i.e., number of model parameters), with the best model having the lowest AIC_c_ score. The level of support for an AIC_c_ value was evaluated by ΔAIC_c_ (ΔAIC_c_ = AIC_*i*_ − AIC_min_). Models with ΔAIC_c_ values of 0–2 have equivalent support as the best model, whereas those with ΔAIC_c_ >2 were not well supported by the data. We used AIC weights (ω_*i*_) to provide a relative measure of evidence that a particular model is the best model for the observed data^73^. The significance of the explanatory terms in models were assessed using likelihood ratio (LR) tests, and estimated marginal means ± SEM and 95% confidence intervals are reported. Model results were used to provide insight into biological and/or non-biological factors associated with observed patterns in hormone measurements, and the value of urea normalization for whale blow. Statistical analyses were performed using the software SPSS® (version 20 for Macintosh, SPSS Inc.).

## Electronic supplementary material

Supplementary information
