# Supplementary material for: Quantifying hormones in exhaled breath for physiological assessment of large whales at sea
Source: Sci Rep. 2018 Jul 17;8:10031. doi: 10.1038/s41598-018-28200-8 (PMC6050234; doi:10.1038/s41598-018-28200-8)
Supplement: Supplementary file 1 — Supplementary information [file 41598_2018_28200_MOESM1_ESM.docx]

**SUPPLEMENTARY INFORMATION**

**Quantifying hormones in exhaled breath for physiological assessment of large whales at sea**

Elizabeth A. Burgess^1*^; Kathleen E. Hunt^2^; Scott D. Kraus^1^; Rosalind M. Rolland^1^

^1^*Anderson Cabot Center for Ocean Life, New England Aquarium, Boston, MA 02110*

^2^*Center for Bioengineering Innovation, Northern Arizona University, Flagstaff, AZ 86011*

*Corresponding author: [eburgess@neaq.org](mailto:eburgess@neaq.org)

**METHODS:**

***Assay verification***

1. **Parallelism test**

Aliquots of whale blow extract samples were pooled, and then serially diluted from neat (undiluted; 1:1) through 1:2, 1:4, 1:8, 1:16, 1:32, 1:64. Parallelism was tested by assaying the serial dilutions of blow extract pool alongside known-dose analyte (i.e., urea, progesterone, testosterone, cortisol) standards, following published guidelines^1^. Optical density (or percent binding for hormone assays) was modeled against relative dose, with the strongest dilution assigned a nominal value and each subsequent dilution assigned a relative dose of ½ that of the previous dilution. Parallelism testing of urea assay for whale blow extracts has not previously been performed (shown in Supplementary Figure 1a); whereas, successful parallelism results for various hormone assays have already been published for right whale blow^2^.

The resulting binding curves were assessed with an *F* test for difference of slope, with the linear portion of the blow extract dilution curve compared to the standard curve that had been assayed in the same run determination ^1^. In parallelism testing, the desired result is a lack of significance (*P* values > 0.1), providing strong evidence that the dilution curves and standard curve were parallel to each other. Parallelism of these curves supports the assumption that binding characteristics allow for the reliable determination of analyte levels in diluted whale blow extracts.

**2. Accuracy (matrix effect) test**

Accuracy testing was performed using dilution of 1:4 for progesterone and testosterone, and neat (1:1) for cortisol and urea assay. These dilutions were selected by consulting parallelism test data for a dilution that fell between 60 and 80% binding (percentage of antibody bound to labelled hormone), a region of the binding curve that typically has good mathematical accuracy while requiring minimal sample volume. For each accuracy test, each standard (i.e., a full standard curve) and blank wells (zero dose) were spiked with an equal volume of appropriately diluted whale blow extract, and assayed in tandem with a second standard curve that was spiked with assay buffer. Parallelism testing of urea assay for whale blow extracts has not previously been performed (shown in Supplementary Figure 1b); whereas, successful parallelism results for various hormone assays have already been published for right whale blow^2^.

Accuracy test results were graphed as apparent (spiked with whale blow extract) vs. known concentration, and assessed using linear regression. The desired result for accuracy testing is a positive linear relationship, with acceptable accuracy defined as *r*^2^ > 0.95 and slope within the range of 0.7–1.3^1^. It was concluded that matrix effects were minimal and that sample diluents of whale blow extract were compatible with the assay kits used in this study.

**(a)** **(b)**

**Supplementary Figure 1**. Validation plots for measuring urea in blow extracts North Atlantic right whales, using colorimetric assay. (a) Close parallelism between serially diluted samples (dilutions 1:1 through 1:64; open circles) to the urea standard curve (0.04–10.0 mg/dL; closed circles) (*P* = 0.15); (b) good accuracy demonstrated by the positive linear relationship of known urea concentration against apparent concentration in spiked samples (solid line; *y* = 0.04 + 0.99*x*), with a slope of near 1.0 (dotted line represents ideal accuracy slope of 1.00, i.e., expected:observed relationship of 1:1).

**RESULTS:**

***Assay interference***

**Supplementary Table 1.** Quantification of exogenous assay interference in negative control samples (i.e., without the biological sample matrix) of dish and nitex mesh sampling devices, measured for each analyte investigated (urea, progesterone, testosterone and cortisol). Absolute levels of each analyte measured in whale blow sample extracts were corrected for known assay interference by subtracting the mean negative control sample concentration for that sampling device from the measured concentration.

|  |  | |  |  |  |  |  |  |  |
| --- | --- | --- | --- | --- | --- | --- | --- | --- | --- |
|  | Analyte | | Assay interference  in negative control samples (Mean ± SEM) | |  | Absolute level in blow samples adjusted for assay interference adjusted for interference | | |  |
|  |  |  | for dish  samplers | for nitex mesh samplers |  | Mean ± SEM | Median | Maximum |  |
|  | Urea (mg/dL extract) | | non-detectable | 0.129 ± 0.053 |  | 0.109 ± 0.016 | 0.057 | 1.041 |  |
|  | Progesterone (ng/mL extract) | | 0.002 ± 0.001 | 0.251 ± 0.040 |  | 0.222 ± 0.040 | 0.113 | 1.841 |  |
|  | Testosterone (ng/mL extract) | | 0.003 ± 0.001 | 0.063 ± 0.010 |  | 0.084 ± 0.016 | 0.041 | 0.899 |  |
|  | Cortisol (ng/mL extract) | | 0.002 ± 0.000 | 0.027 ± 0.002 |  | 0.016 ± 0.003 | 0.004 | 0.174 |  |
|  |  |  | |  |  |  |  |  |  |

***Variation in blow hormones (using absolute values)***

**Supplementary Table 2.** Ranking of *a priori* models explaining the variation in absolute levels of (a) progesterone and (b) testosterone as reproductive hormones, and (c) cortisol as stress-related hormone, measured in the blow of right whales. Biological and sampling variables were all modeled as fixed effects, except for individual whale modeled as a random effect. Models were ranked based on Akaike’s Information Criterion adjusted for small sample size (AIC_c_)_._ The lowest AIC*_c_* indicates the best model for each hormone measured (highlighted in bold). AIC_c_ weights (ω*_i_*) sum to 1 and indicate the relative likelihood of the model.

|  |  |  |  |  |  |  |
| --- | --- | --- | --- | --- | --- | --- |
|  | Model parameters | AIC_c_ | df | ΔAIC*_i_* | *ω*_i_ |  |
|  |  |  |  |  |  |  |
|  | **(a) Absolute progesterone:** |  |  |  |  |  |
|  | **sampling device** | **-133.7** | **74** | **0.00** | **1.00** |  |
|  | sample quality | -83.0 | 73 | 50.74 | 0.00 |  |
|  | sex + individual whale | -76.7 | 74 | 57.02 | 0.00 |  |
|  | individual whale | -76.6 | 75 | 57.14 | 0.00 |  |
|  | sex + age class + individual whale | -76.0 | 73 | 57.79 | 0.00 |  |
|  | age class + individual whale | -75.5 | 74 | 58.21 | 0.00 |  |
|  |  |  |  |  |  |  |
|  | **(b) Absolute testosterone:** |  |  |  |  |  |
|  | **sampling device** | **-265.6** | **82** | **0.00** | **0.93** |  |
|  | sex + individual whale | -259.1 | 82 | 6.53 | 0.04 |  |
|  | sample quality | -257.7 | 81 | 7.86 | 0.02 |  |
|  | sex + age class + individual whale | -257.1 | 81 | 8.47 | 0.01 |  |
|  | individual whale | -255.3 | 83 | 10.32 | 0.01 |  |
|  | age class + individual whale | -253.1 | 82 | 12.46 | 0.00 |  |
|  |  |  |  |  |  |  |
|  | **(c) Absolute cortisol:** |  |  |  |  |  |
|  | **sampling device** | **-656.8** | **86** | **0.00** | **1.00** |  |
|  | sample quality | -586.9 | 85 | 69.94 | 0.00 |  |
|  | time of day + individual whale | -581.5 | 75 | 75.30 | 0.00 |  |
|  | age class + individual whale | -581.0 | 85 | 75.76 | 0.00 |  |
|  | repeat sample + individual whale | -580.2 | 84 | 76.63 | 0.00 |  |
|  | sex + individual whale | -579.0 | 85 | 77.76 | 0.00 |  |
|  | individual whale | -578.4 | 86 | 78.42 | 0.00 |  |
|  | sampling duration + individual whale | -574.1 | 84 | 82.74 | 0.00 |  |
|  |  |  |  |  |  |  |
|  |  |  |  |  |  |  |

**REFERENCES:**

1. Grotjan, H. E. & Keel, B. A. in *Immunoassay* (eds. Diamandis, E. P. & Christopoulos, T. K.) 51–93 (Academic Press, 1996).

2. Hunt, K. E., Rolland, R. M. & Kraus, S. D. Detection of steroid and thyroid hormones via immunoassay of North Atlantic right whale (*Eubalaena glacialis*) respiratory vapor. *Mar. Mamm. Sci.* (2014).
